# Evaluating the Effect of Packaging Materials on Extra Virgin Olive Oil Quality Under Simulated Household Use and Storage Conditions

**DOI:** 10.3390/foods15111948

**Published:** 2026-06-01

**Authors:** Beatrice Sordini, Stefania Urbani, Roberto Selvaggini, Agnese Taticchi, Maurizio Servili, Ilenia Dottori, Gianluca Veneziani, Franco Famiani, Arianna Bonucci, Davide Nucciarelli, Sonia Esposto

**Affiliations:** 1 Department of Agricultural, Food and Environmental Sciences, University of Perugia, Via S. Costanzo, 06126 Perugia, Italy; beatrice.sordini@collaboratori.unipg.it (B.S.); stefania.urbani@unipg.it (S.U.); roberto.selvaggini@unipg.it (R.S.); agnese.taticchi@unipg.it (A.T.); maurizio.servili@unipg.it (M.S.); gianluca.veneziani@unipg.it (G.V.); franco.famiani@unipg.it (F.F.); arianna.bonucci@unito.it (A.B.); davide.nucciarelli@gmail.com (D.N.); sonia.esposto@unipg.it (S.E.); 2 Department of Agricultural, Forestry and Food Science, University of Turin, Via Verdi 8, 10124 Turin, Italy

**Keywords:** extra virgin olive oil, real-time shelf life, packaging materials, bag-in-box, chrome-plated tin, antioxidants, volatile compounds, sensory analysis, household storage

## Abstract

Extra virgin olive oil (EVOO) is prone to oxidative degradation during storage, especially after opening, when exposure to oxygen and light accelerates the depletion of antioxidants and promotes the formation of oxidation products, including hydroperoxides and C_7_–C_9_ aldehydes associated with rancidity. Packaging materials play a critical role in preserving EVOO quality in real-use conditions. This study comparatively evaluated the effectiveness of three widely used packaging materials: green polyethylene terephthalate (PET), bag-in-box (BB), and chrome-plated tin (CPT) in preserving regulatory, sensory, and health-related qualities of EVOO under simulated household use and storage conditions. Methods: PET, BB, and CPT containers (3 L) were totally filled with the same EVOO and stored at 25 °C under a 12 h light/dark cycle, with 75 mL withdrawn daily for 40 days to mimic consumer use. Every 10 days, regulatory quality indices (free acidity (FA), peroxide value (PV), K_232_, and K_270_), antioxidants, volatile compounds, and sensory attributes were evaluated. Results: Free acidity, K_232_, and K_270_ increased slightly in EVOOs across all containers, while EVOO stored in PET showed a sharp rise in PV, exceeding the legal limit after 30 days. Antioxidant depletion was significantly (*p* ≤ 0.05) more pronounced in the EVOO stored in PET (44% α-tocopherol and 26% total phenols, respectively) than in BB (1% and 11%) and CPT (5% and 12%). The EVOO stored in PET also showed a reduction in C_5_–C_6_ aldehydes (−26% and −24% compared to BB and CPT, respectively), alongside an accumulation in C7–C9 aldehydes (+8% and +5%), exhibiting higher loss of C_5_–C_6_ aldehydes and of C_7_–C_9_ aldehydes, which is responsible for fruity–green notes and rancid defect, respectively, compared to BB and CPT. Conclusions: Overall, the EVOOs stored in BB, followed by CPT, showed higher oxidative stability than those stored in PET, resulting in prolonged “extra virgin” classification and improved preservation of antioxidant content, volatility profile, and sensory quality during consumer-level storage.

## 1. Introduction

Among vegetable oils, extra virgin olive oil (EVOO) is much appreciated by consumers for its high content of monounsaturated fatty acids and its richness in minor compounds, including phenolic and volatile compounds, which are mainly responsible for its biological and sensory properties [1]. Since 2011, the European Food Safety Authority (EFSA) has endorsed a health claim stating that EVOO polyphenols (particularly those standardised in terms of hydroxytyrosol and its derivatives) help protect low-density lipoprotein (LDL) particles from oxidative damage [2]. This claim refers to the daily consumption of EVOO providing at least 5 mg of hydroxytyrosol (including derivatives) per 20 g of olive oil [2,3]. Despite these healthy properties, like other vegetable oils and fats, EVOO undergoes progressive decline in sensory attributes, health-promoting effects, and regulatory indices during storage. Oxidation is the main cause of this deterioration, as it leads to breakdown of the endogenous antioxidants and accumulation of undesirable compounds in EVOO. This process occurs during the primary oxidation stage—such as the formation of hydroperoxides—and advances to the secondary oxidation stage, which involves compounds such as aldehydes, ketones, and other volatile compounds that are associated with sensory defects [4,5]. Consequently, consumer acceptability of EVOO diminishes over time [4,5,6]. The susceptibility of EVOO to oxidation is determined not only by its chemical composition (fatty acid composition, tocopherol, and secoiridoid derivatives) [7,8,9,10] but also by environmental factors [11], including the light exposure [10], temperature [12,13], oxygen availability [14], and presence of activators (chlorophylls and transition metals) [4,5]. In particular, chlorophylls affect both the colour and oxidative stability of EVOO. In the absence of light, they act as antioxidants, whereas under light exposure, they behave as pro-oxidants, promoting photo-oxidation, which proceeds faster than autoxidation. This dual role highlights the importance of chlorophyll content and explains why olive oil, compared to other vegetable oils, requires protection from light to preserve its quality [4]. Therefore, packaging represents a key factor in preserving EVOO’s qualitative standards since the container can either accelerate or delay degradation by oxidation, depending on its ability to limit gaseous permeability and provide UV–visible light protection [15,16,17,18,19,20,21,22].

Over the years, several packaging materials have been proposed and tested to extend the shelf life of EVOO, from conventional glass bottles [15,16,19] to plastics [19,20,23], metals [19,24], and, more recently, multilayer systems [24,25,26,27] or innovative bioplastics [28]. Glass, especially in dark or UV-resistant forms, guarantees gas impermeability, while still allowing some photooxidative stress, depending on its UV–visible light-blocking efficiency [15,16,27]. Plastics such as PET are attractive for their ease of use and low costs but have lower barrier capacity and may promote migration phenomena [20,23]. Other plastic materials, such as polyethylene (PE) and polypropylene (PP), typically offer less effective protection due to their high oxygen permeability [29]. In contrast, alternative packaging materials, such as bag-in-box (BB), have demonstrated promising results in extending shelf-life preservation [24,25]. Given the limitations of accelerated tests, increasing attention has been paid to real-time shelf life studies [20]. In this context, Esposto et al. systematically investigated the natural decline of EVOOs with different antioxidant levels (high and medium) under storage conditions closer to real market scenarios, including light exposure [10] or darkness [9], using different packaging materials [27]. These studies demonstrated that EVOOs with higher antioxidant content exhibited greater oxidative stability, while packaging and storage conditions (light vs. dark, oxygen exposure) strongly affected the rate of quality deterioration, highlighting the protective role of appropriate containers in preserving both chemical and sensory characteristics. The impact of packaging materials has been further confirmed by several studies. Méndez and Falqué [19] reported a progressive decrease in EVOO quality during 6 months of storage, particularly in those bottled in plastic and glass bottles, whereas tin and Tetra Brik^®^ (Tetra Pak International S.A., Lund, Sweden) provided the best preservation. Similarly, Abuhabib et al. [30] and De Leonardis et al. [26] demonstrated the effectiveness of bag-in-box systems in retaining the phenolic compounds and sensory quality of EVOO compared to stainless steel with inert headspace [30] even under conditions simulating repeated oil withdrawal [26]. Other studies have explored innovative solutions: Farris et al. [28] tested the performance of two innovative packaging types (transparent films with UV blockers and metallised plastics); they outperformed traditional brown-amber glass bottles in preserving the phenolic content and aromatic profile during accelerated storage conditions. Meanwhile, Zarazir et al. [31] reported that polylactic acid (PLA)-based materials provided better protection than conventional plastics, although glass remains among the most effective options.

Extensive research has examined the effects of retail shelf storage conditions on the quality of EVOO packaged in different materials, mainly focusing on long-term and primary shelf life (the period before the package is opened). However, limited information is available on EVOO behaviour after opening under domestic storage conditions (secondary shelf life (SSL), known as the period during which the product maintains acceptable quality characteristics once opened [32]. During this time, the headspace progressively increases as oil is consumed [14,33,34]. Under these conditions, repeated opening, handling, and bottle shaking facilitate oxygen transfer into the headspace, and simultaneous light and oxygen exposure accelerate oxidative degradation [32]. A household-oriented study on Cobrançosa EVOO [14] highlighted the quality decrease after bottle opening and proposed predictive models for consumer-level storage. Despite these efforts, systematic comparisons among commonly used commercial packaging systems under simulated household conditions are still scarce, particularly for bag-in-box systems, green PET, and chrome-plated tin (CPT) [24,26]. Therefore, this study aimed to investigate the influence of three different packaging systems on overall EVOO quality during post-opening storage (secondary shelf life) under conditions simulating real household use, considering the specific functional characteristics of each packaging system.

## 2. Materials and Methods

### 2.1. Materials

Pure analytical standards of volatile and phenolic compounds such as tyrosol (p-HPEA) and hydroxytyrosol (3,4-DHPEA), vanillic acid, caffeic acid, and α-tocopherol were supplied by Merck (Milan, Italy). The isomers of oleuropein aglycon (3,4-DHPEA-EA), oleocanthal (p-HPEA-EDA), oleacein (3,4-DHPEA-EDA), and lignans ((+)-1-acetoxypinoresinol and (+)-pinoresinol) were extracted from an EVOO, as reported by Selvaggini et al. [35]. For chromatographic analysis, methanol, n-hexane, 2-propanol, and glacial acetic acid, all HPLC-grade, were purchased from VWR (Milan, Italy).

The EVOO used in this study was produced from a single batch of Moraiolo olives grown in organic orchards in the Umbria region, near Assisi (Italy). Olives were harvested in mid-October during the 2019/2020 crop season and processed within 24 h via mechanical extraction using a three-phase system. This EVOO was kindly supplied by Le Mandrie farm (Assisi, Italy). At the time of packaging (time zero, T0), the EVOO was analysed to assess its chemical composition and sensory profile and to determine its classification as “extra virgin” [36] (Table 1).

### 2.2. Simulation of EVOO Household Storage/Consumption Conditions

To assess the impact of packaging material on the quality parameters of extra virgin olive oil (EVOO) under simulated household conditions, an identical 9 L EVOO sample was divided into three 3 L aliquots. These aliquots were then packaged in three different primary packaging, each with a capacity of 3 L. Since direct measurements of ultraviolet–visible (UV–Vis) light transmittance, oxygen permeability, and water vapour permeability of different packaging were not performed in this study, these properties were estimated based on literature data and typical values reported for similar materials. All packaging systems were purchased from a local supermarket in Perugia, Italy. Theoretically, the technical specifications of the EVOO packaging materials were as follows: (i) A matte green food-grade polyethylene terephthalate (PET) container (hereafter referred to as “packaging PET”; Figure S1a) with an oxygen permeability, defined as the rate at which O_2_ passes through the material normalised by thickness and pressure, of 0.055 cm^3^·m^−2^·day^−1^·atm^−1^ [29], provided moderate water vapour barrier properties and partial protection against UV–visible light due to the green pigmentation [29]. (ii) A bag-in-box (BB) system consisted of a multilayer pouch placed inside a parallelepiped-shaped, corrugated paperboard box (hereafter, “packaging BB”; Figure S1b). The pouch was made of a multilayer plastic material, with a layered structure of PET (external)/adhesive/metallised PET/adhesive/LDPE (internal), reported to provide very low oxygen permeability (<0.005 cm^3^·m^−2^·day^−1^·atm^−1^ [25]), low water vapour permeability, and high barrier properties due to the metallised layer and external paperboard box [25,26]. (iii) A tin-plated parallelepiped can, with the internal varnish based on chrome-plated stripe coating (hereafter, “packaging CPT”; Figure S1c), is characterised by negligible oxygen permeability through the metal wall and complete protection from light, along with very low water vapour permeability [25,26]. The oxygen permeability values were derived from literature sources and refer to standardised conditions (23 °C and 0% relative humidity) according to established methods [37]. Each container was filled and placed on a shelf to undergo a 40-day real-time shelf-life test under light exposure conditions of 500 lux for 12 h per day at a temperature of 25 °C. An electronic device monitored the duration and intensity of light exposure. Each day, all containers were opened, and 75 mL of EVOO was withdrawn, gradually increasing headspace and EVOO’s exposure to oxygen. This effect was observed in PET and CPT containers, while in the BB system, the bag collapsed as oil was dispensed through the valve. Although oxygen levels were not directly measured, the experimental setup mimicked typical household conditions, including repeated opening, air exposure, and light exposure, rather than ideal storage in an inert atmosphere or darkness. The withdrawal volume was intended to approximate household consumption, based on EFSA’s recommended daily olive oil intake (around 20 g per person) [2] and assuming a household with multiple members, though actual consumption habits may vary. Under these conditions, the 3 L containers were emptied in 40 days, enabling the assessment of quality changes during the secondary shelf life after opening.

The experimental procedure was replicated under identical conditions using the same EVOO batch to obtain samples for sensory analysis. Every ten days, the collected EVOO was subjected to chemical and sensory analyses as described in the following subsections. Before analysis, the samples were stored at 12 °C in the dark. The analyses ended after forty days of storage, with samples collected at 10, 20, 30, and 40 days.

### 2.3. Analytical and Sensory Evaluations of EVOO

For each EVOO sample (PET, BB, and CPT), all chemical analyses were performed in two independent determinations (*n* = 2) at each sampling time (0, 10, 20, 30, and 40 days), and the results are expressed as mean ± standard deviation (SD).

#### 2.3.1. Regulatory Quality Indices of EVOO

EVOO quality characteristics, including free acidity (FA), peroxide value (PV), and specific extinction coefficients (K_232_, K_270_, and ∆K), were assessed in accordance with the official methods of the Commission Delegated Regulation (EU) 2022/2104 [36].

#### 2.3.2. Evaluation of Antioxidants and Estimation of the EFSA Health Claim of EVOOs

For α-tocopherol content, approximately 1 g of EVOO was diluted in 10 mL of n-hexane, filtered through a 5-μm PVDF syringe filter (Whatman, Clifton, NJ, USA), and analysed via HPLC equipped with a diode array detector and fluorescence detector (HPLC-DAD-FLD, Agilent Technologies, Santa Clara, CA, USA), following the in-house procedures validated in a previous study [38].

For hydrophilic phenols, the extraction and HPLC analysis with diode array detection (Agilent Technologies, Santa Clara, CA, USA) were performed in accordance with the methodology described and in-house validated by Selvaggini et al. [35], employing a Spherisorb ODS-1 column 250 mm × 4.6 mm with a particle size of 5 μm (Waters, Milford, MA, USA). The HPLC equipment and analytical conditions were consistent with those previously described. Phenolic compounds were quantitatively evaluated using response factors specific to each compound. Notably, the response factor for oleocanthal was used to assess ligstroside aglycone quantitation, as structurally related compounds, due to the absence of a specific standard. The results are expressed as mg kg^−1^ of EVOO.

#### 2.3.3. HS-SPME-GC–MS Analysis of EVOO Volatile Compounds

The volatile compounds in EVOO were analysed using HS-SPME-GC-MS, following the protocol described by Taticchi et al. [39]. Briefly, for sample preparation, about 6 g of EVOO was mixed with 50 μL of isobutyl acetate (internal standard at a final concentration of 9.8 mg kg^−1^ in oil) and vortexed for 1 min at 2500 rpm. Then, 3 g of the homogenised sample was transferred into a 20 mL headspace vial. The volatile compounds were extracted using a 2 cm Stableflex SPME fibre coated with 50/30 μm divinylbenzene/Carboxen/polydimethylsiloxane (DVB/Carboxen/PDMS; Supelco, Inc., Bellefonte, PA, USA). Samples were equilibrated by shaking for 10 min at 35 °C; then, the fibre was exposed to the headspace for 30 min at 35 °C. Analytes were thermally desorbed in the GC injector set in splitless mode for 5 min at a temperature of 250 °C. GC–MS analyses were carried out using a system consisting of an Agilent 7890B GC equipped with a Multimode Injector (MMI) 7693A (Agilent Technologies, Santa Clara, CA, USA) and a PAL3 RSI 120 autosampler (CTC Analytics AG, Zwingen, Switzerland), coupled to a mass spectrometer Agilent 5977B single quadrupole with an EI XTR source (Extractor Ion Source) (Agilent Technologies, Santa Clara, CA, USA). Separation used a DB-WAXetr capillary column (50 m × 0.32 mm, 1 μm) (Agilent Technologies, Santa Clara, CA, USA) with helium at 1.7 mL/min. The oven started at a temperature of 35 °C for 4 min, which then rose to 45 °C at 5 °C/min, 150 °C at 4 °C/min, 180 °C at 8 °C/min for 2 min, and 210 °C at 11 °C/min for 13.77 min, totalling 55 min. The transfer line temperature was set at 215 °C, while the MS conditions were for source and quadrupole 190 °C and 150 °C, respectively. Mass spectra were acquired in electron ionisation (EI) mode at 70 eV, covering a mass range of 25–350 *m*/*z* at a scan rate of 4.3 scan/s. Measurements were taken in both full-scan and selected ion monitoring (SIM) modes. Data acquisition and analysis were performed using Agilent MassHunter software version 10.1 (Agilent Technologies, Santa Clara, CA, USA). Volatile compounds were identified based on a comparison of their mass spectra and retention times with those of pure analytical standards and via comparison with the NIST-2014 library spectra. The volatile compound quantification was expressed as μg kg^−1^ of oil, with calibration curves constructed for each compound using the internal standard method.

#### 2.3.4. Sensory Analysis of EVOO

In accordance with the ISO 8586:2023 standard [40] and the official procedures outlined in [41], a quantitative descriptive sensory analysis (QDA) was conducted by a panel of 10 experienced judges. This panel, consisting of four men and six women aged 30 to 50, was selected from the academic staff of the Department of Agricultural, Food and Environmental Science at Perugia University. Prior to participating in the sensory analysis, all panellists were informed of the ethical standards outlined in the WMA Declaration of Helsinki and provided their consent. Five sensory evaluation sessions were held at different sampling intervals (0, 10, 20, 30, and 40 days) in individual booths within a controlled environment maintained at 22 °C under standard white tube lighting. A modified profiling sheet was used, encompassing both positive attributes and off flavours [27]. The attributes evaluated included green, yellow, fruity, cut grass/herbaceous, green apple, hay-like, floral, almond, artichoke, tomato, sweet, bitter, pungent, earthy, winey-vinegary, fusty, and rancid. Each attribute was evaluated using a 9 cm unstructured line scale anchored at 0 (absence of the attribute) and 9 (very intense). For all attributes, the coefficient of variation did not exceed 20% based on the average ratings from the 10 panellists, indicating a satisfactory level of agreement among judges and supporting the consistency of the sensory evaluation [41]. A sensory defect was considered perceptible when it was consistently detected (non-zero intensity) by at least 50% of the panellists over the storage period [41]. For sensory evaluation, residual EVOO from chemical analyses was pooled and homogenised with the samples collected for sensory evaluation and on the previous sampling day to obtain a representative composite sample (approximately 160 mL) for each evaluation step. This approach was adopted to obtain a sufficient volume for sensory evaluation and to ensure that the sample was representative of the same EVOO conditions during the storage period. The sensory evaluation was therefore performed on composite samples representing each storage interval. The EVOO samples were served in white plastic tasting glasses, labelled with three-digit random codes, and presented in a balanced order.

### 2.4. Statistical Analysis

The chemical and sensory data were analysed using both uni- and multivariate methods. All chemical analyses were performed in two independent determinations (*n* = 2), and the results are expressed as mean ± standard deviation. Quantitative descriptive sensory data at each time point are shown as the mean of 10 experienced panellists’ sensory evaluations. Data were analysed via one- and two-way analysis of variance (ANOVA) using SigmaPlot v. 12.3 software (Systat Software Inc., San Jose, CA, USA). Two-way ANOVA was applied to antioxidant compounds (α-tocopherol and hydrophilic phenols) to assess the effects of packaging material and storage time, treated as fixed factors, and their interaction (Table S2). When significant effects were detected (*p* ≤ 0.05), Tukey’s post hoc test was used for multiple comparisons. The SIMCA 13.0 chemometric package (Umetrics AB, Umeå, Sweden) was used to carry out principal component analysis (PCA) and partial least squares projections to latent structures (PLS) on the chemical and sensory data. Both models were built using the mean values of all variables for each sample and storage time. For PCA and PLS analyses, all variables were normalised and autoscaled to unit variance. Cross-validation was used to determine the number of significant components, and only these validated components were considered for model interpretation, to reduce the risk of overfitting.

## 3. Results and Discussion

### 3.1. Evaluation of EVOO Quality at Time of Packaging (T0)

At the time of packaging (T0), EVOO was analysed across three containers (PET, BB, and CPT) to assess its regulatory, chemical, and sensory characteristics. As shown in Table 1, free acidity, peroxide value (PV), and spectrophotometric indices (K_232_, K_270_, and ΔK) were significantly lower than the EU legal thresholds for the “extra virgin” category [36]. The EVOO was characterised with a rich antioxidant profile (173.8 ± 0.6 mg kg^−1^ of α-tocopherol and 956 ± 3.9 mg kg^−1^ total hydrophilic phenols, with oleuropein derivatives accounting for approximately 83%, ligstroside derivatives for about 11%, and a minor contribution from lignans). This composition should be considered when interpreting the oxidative stability observed during storage, as the oxidative behaviour of EVOO was strongly affected by its initial phenolic heritage and concentration [10,27]. The EVOO volatile fraction, determined via HS-SPME/GC–MS, revealed a complex and abundant aromatic profile dominated by (E)-2-hexenal, hexanal, cis-3-hexenol, hexanol, (E)-2-hexenol, hexyl acetate, and 1-penten-3-one, which is consistent with fresh green–fruit notes [42,43,44,45]. Sensory analysis confirmed the absence of defects and highlighted strong bitter and pungent attributes that are typical characteristics of high-quality EVOO [45,46,47,48,49].

### 3.2. Evolution in EVOOs Chemical and Sensory Quality During Simulated Household Use and Storage

**Figure 1:**
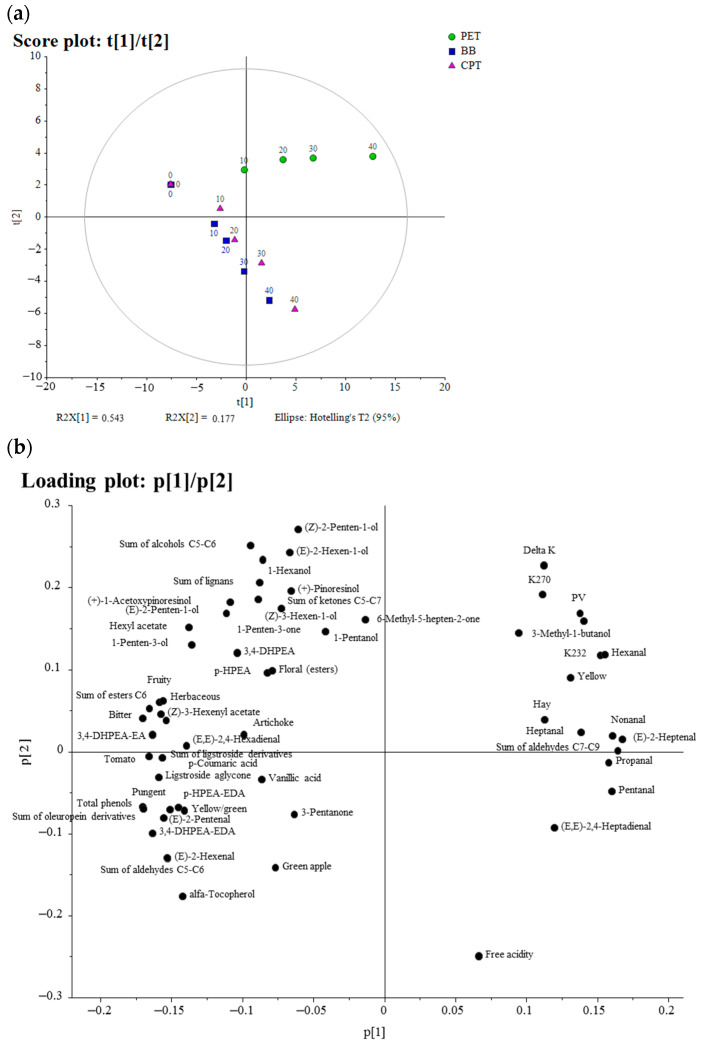
Score (**a**) and loading (**b**) plot of the first two principal components of the PCA model built using mean values of EVOOs stored in three different packaging materials, considering all analytical and sensory variables.

A preliminary PCA model was built using both analytical and sensory variables to explore the effect of packaging materials on EVOO quality during household use and storage over 40 days (Figure 1). The PCA model explains 87% of the dataset’s total variance, with four significant principal components (PCs) accounting for 54%, 18%, 9% and 6%, respectively. The Q^2^ value was 63%, indicating good predictive capability. In the score plot (t_1_/t_2_) of the first two PCs (Figure 1a), the EVOOs were distinctly clustered according to the packaging material along PC1 (moving from left to right on the score). The EVOO samples were also significantly discriminated by storage time along PC2 (from top to bottom, Figure 1a). In particular, the EVOOs stored in BB were clustered in the lower-left quadrant of the plot, while those stored in CPT were positioned nearby. In contrast, the PET-stored EVOOs were positioned in the upper-right quadrant, exhibiting a distinct separation from the BB and CPT EVOOs. As storage time increased from 0 to 40 days, the EVOOs packaged in BB moved downward and to the left, reflecting gradual changes. The samples stored in CPT fell between those in PET and BB, mainly in the lower part of the plot. Over time, EVOO samples stored in CPT also shifted downward, following a similar trend to that of BB but with less compact movement, suggesting greater changes. Moreover, EVOOs stored in PET remained distinctly separated from the others, located on the right side, and exhibited variation along PC2 as storage days increased. The relative loading plot (p_1_/p_2_) (Figure 1b) indicates that the variables with the greatest influence on sample distribution along PC1 were those associated with EVOO quality and freshness, including total phenols, oleuropein derivatives, tomato, fruit, sum of aldehydes C_5_–C_6_, 1-penten-3-ol, (E)-2-hexenal, 3,4-DHPEA-EDA, (E)-2-pentenal, pungent, ligstroside aglycone, (Z)-3-hexenyl acetate, and hexyl acetate, which were characteristic of the fresh samples (time 0) located on the left side of PC1 on the score plot. This is consistent with the literature, where the sensory attributes of tomato leaf and fruit are strongly correlated with volatile compounds produced through the LOX pathway ((E)-2-hexenal and (Z)-3-hexen-1-ol, in particular), as reported by Genovese et al. [44]. Conversely, variables with high positive loadings on PC1 were mainly oxidation-related parameters, including E)-2-heptenal, nonanal, sum of aldehydes C_7_–C_9_, hexanal, PV, K_232_, and yellowing, which increased during storage and characterised the more oxidised samples, particularly those stored in PET for 20 to 40 days, and EVOOs stored in BB and CPT at the end of storage, all clearly contrasted with the fresh samples. This distribution confirms the evolution from fresh to oxidised EVOOs along PC1 during storage time. Along PC2, positive scores were predominantly observed for EVOOs at the initial storage time (T0), which exhibited higher concentrations of the sum of C_5_–C_6_ alcohols ((Z)-2-penten-1-ol, (E)-2-hexen-1-ol, and 1-hexanol) and ΔK. Meanwhile, negative scores correspond to samples stored for longer periods, where free acidity and α-tocopherol had a greater influence on the sample distribution.

**Figure 2:**
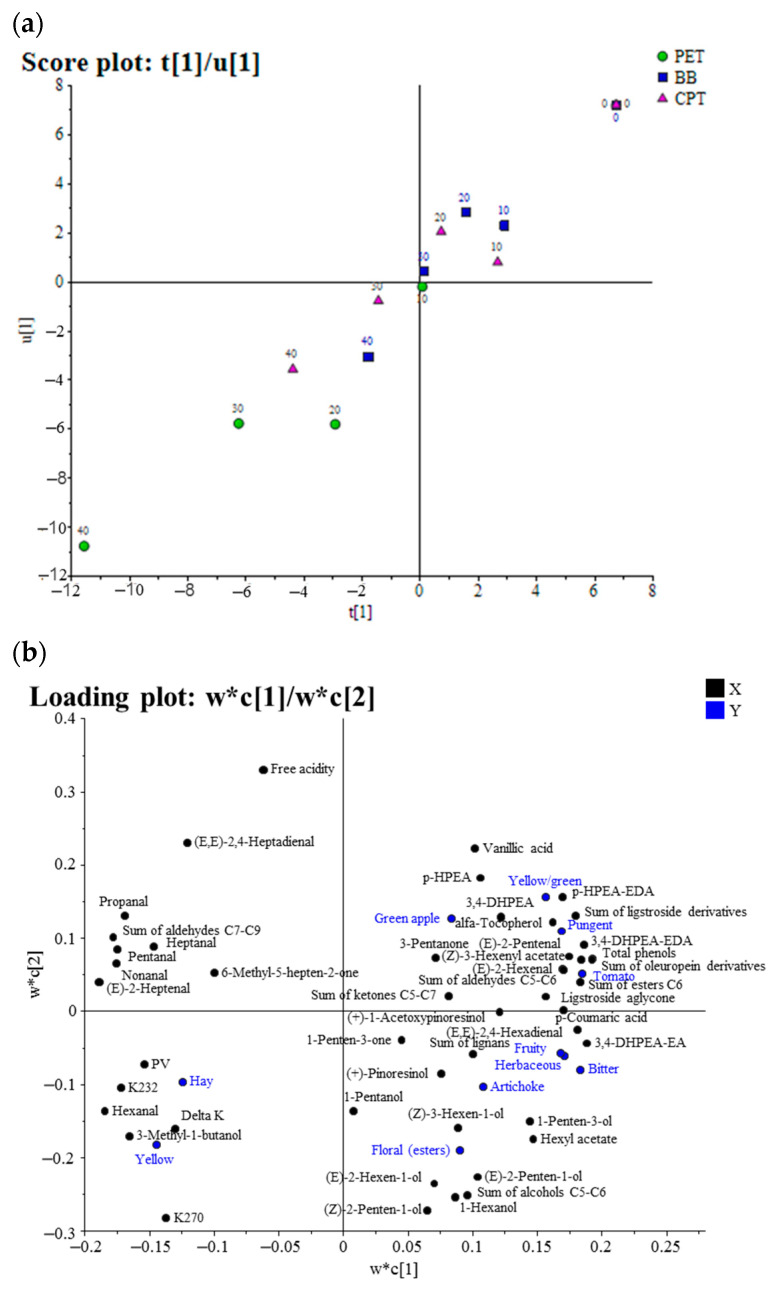
Score plot (t[1]/u[1]) (**a**) and loading plot (w*c[1]/w*c[2]) (**b**) of the PLS model built using mean values of EVOO samples stored in three different types of packaging, considering all analytical (X) and sensorial (Y) variables.

A PLS regression model was built using all analytical (X) and sensory (Y) variables. The model, with three latent variables, accounted for 74% of the total variance in Y and 82% in X, with an overall Q^2^ value of 59%, and the first latent variable explained a large part of the variance (57% in Y and 54% in X). The score plot (t[1]/u[1]) (Figure 2a) demonstrates a strong correlation between the analytical and sensory datasets, clearly illustrating the temporal progression of the EVOO samples during storage. The EVOO samples gradually migrated toward the lower-left quadrant of the plot as storage time increased, indicating deterioration in both physical–chemical and sensory qualities. Among the various packages, EVOOs stored in PET showed the most significant variation along the first latent variable, thereby confirming their heightened susceptibility to quality degradation over time. This trend is consistent with PCA results (Figure 1) and is in good agreement with previous findings reported by Esposto et al. [27]. The loading plot (w*c[1]/w*c[2]) (Figure 2b) depicts the relationships between the analytical data and sensory attributes. The first latent variable primarily distinguished EVOO samples by markers of oxidative degradation (e.g., elevated peroxide value and K_232_), which correlate with a decline in positive sensory descriptors such as fruity, artichoke, and tomato. Conversely, samples located on the positive side of t[1] correspond to fresher oils characterised by higher intensities of positive sensory notes and lower levels of oxidation-related parameters [44,45,46,47,48,49].

These data closely align with previous findings by Esposto et al. [10,27,38], demonstrating that PCA and PLS models identify similar positive correlations with VOO storage duration and parameters like K_270_ and C_7_–C_11_ aldehydes, including (E,E)-2,4-decadienal and (E)-2-decenal, as well as polyphenol oxidation products.

### 3.3. Evolution of Regulatory Quality Indices of EVOOs During Simulated Household Use and Storage

**Figure 3:**
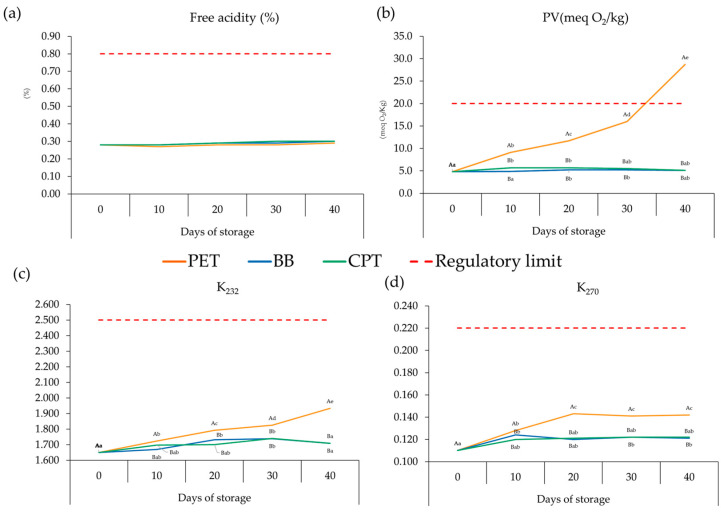
Evolution of regulatory quality parameters of EVOOs stored within three different types of packaging under simulated household use and storage conditions over 40 days: (**a**) free acidity; (**b**) PV; (**c**) K_232_; and (**d**) K_270_. The dashed red line indicates the maximum values of olive oil quality parameters for EVOO quality-grade classification [36]: free acidity, ≤0.8% oleic acid; PV, ≤20 meq O_2_/kg; K_232_, ≤2.50; K_270_, ≤0.22. Results are expressed as the mean ± standard deviation of two independent determinations (*n* = 2). Uppercase letters indicate significant differences (*p* ≤ 0.05) among different EVOOs packaging at the same storage time. Lowercase letters indicate significant differences (*p* ≤ 0.05) within the same EVOO packaging during storage.

To simulate typical household usage, all containers were opened each day, and an aliquot of EVOO was taken. This procedure allowed air to enter the headspace at each opening, progressively increasing oxygen exposure and promoting oxidative and hydrolytic degradation processes, thus reproducing realistic domestic storage conditions where repeated opening, headspace formation, and exposure to oxygen and light occur simultaneously during product use [34]. These processes lower the EVOO’s marketable value and sensory attributes, leading to the generation of off-flavours and harmful reactive species, such as free radicals [22,33,34]. To assess the impact of the various packaging materials on reducing EVOO oxidative processes, the regulatory quality parameters (free acidity (FA), peroxide value (PV), and spectrophotometric indices (K_232_, K_270_, and ΔK)) were monitored over a period of 40 days under controlled oxidative conditions such as exposure to light and oxygen availability (Table 2 and Figure 3).

Across all packaging containers, FA remained within the limits established for the “extra virgin” category [36]. The FA values of EVOOs consistently stayed well below the threshold of 0.8% oleic acid, with only negligible daily increases (0.0003–0.0006%). Correlation coefficients ranged between 0.45 and 0.90, confirming that hydrolytic degradation was limited and largely independent of packaging type, except for EVOOs stored in BB and CPT. Studies conducted by Cecchi et al. [20], Sanmartin et al. [13], and Rodrigues et al. [33] reported that free acidity remained predominantly stable throughout storage and was minimally affected by packaging, thereby supporting our findings. In contrast, the PV showed the most significant (*p* ≤ 0.05) change as a function of packaging and storage time. The EVOOs stored in PET containers exhibited a rapid increase in PV (R^2^ = 0.90; rate = 0.547 meq O_2_ kg^−1^ day^−1^), exceeding the legal threshold of 20 meq O_2_ kg^−1^ after approximately 30–40 days of storage. As a result, these EVOOs no longer met the standards for the “extra virgin olive oil” category and were declassified as lampante olive oil (LOO), which is not suitable for consumption in its current form and must be refined to make it appropriate for edible use [36] (Figure 3b). Under the experimental conditions, the faster oxidative degradation observed in EVOO stored in PET probably suggests lower overall protective performance, although the individual contributions of oxygen barrier properties and light transmission were not separately assessed. Similarly, Alimi et al. [14] observed a sufficient PV increase for declassification to LOO [36] after 35 days of storing an EVOO in amber glass bottles with light exposure under household conditions. Samples stored in BB and CPT packaging exhibited slower oxidation rates than those stored in PET. A negligible increase in PVs was observed, with a 6.3% rise for both EVOOs stored in BB and CPT compared to the initial values. Lolis et al. [25] reported that light-protective packaging systems, including bag-in-box and tin-plated steel, effectively maintained PV within regulatory limits, except for oils stored in tin-plated steel at 37 °C, which exceeded the EU threshold after 100 days [36].

The specific extinction coefficients K_232_ and K_270_ measure the indices of conjugated fatty acid dienes and trienes, respectively, and are used as indicators of primary and secondary oxidation products in EVOO in this study; differences in these coefficients were observed among the three packaging types used to store EVOO (Table 2 and Figure 3c,d). For K_232_, the strongest correlation with storage time was found in EVOOs stored in PET (R^2^ = 0.98), followed by BB (R^2^ = 0.91) and CPT (R^2^ = 0.67) (Table 2). As discussed for the PV, PET packaging showed a faster rate of increase in K_232_ than BB and CPT, indicating that primary oxidation occurred more rapidly within PET containers. Similarly, K_270_ exhibited higher R^2^ values for BB (R^2^ = 0.95) and CPT (R^2^ = 0.94) than for PET (R^2^ = 0.74). The increase rate was highest in PET (0.00077), while BB (0.00003) showed the lowest, confirming its better protection against the secondary oxidation process. After 40 days of simulated household use and storage conditions, the increases in K_232_ and K_270_ values were lower for EVOOs in BB (1.2% and 10.0%) and CPT (3.1% and 1%) than for PET (12.2% and 29.1%), indicating that none of the samples exceeded the EU limits for EVOO classification [35]. Furthermore, the ΔK values remained largely unchanged (R^2^ < 0.6) throughout the experiment. These findings highlight that oxygen permeability and light transmission are crucial factors influencing the original EVOO’s characteristics; moreover, the PET packaging, which provides a lower protective barrier against light and oxygen, accelerated both primary and secondary oxidation processes compared to BB and CPT containers. Our findings concur with those of previous studies on various factors, including storage duration, light exposure, oxygen levels, temperature, and conditions during storage and transportation, thereby emphasising the importance of packaging in preserving the quality of EVOO [14,25,27,32]. Gambacorta et al. [50] compared the performance of PET bottles with and without an oxygen-active barrier for EVOOs stored in darkness at room temperature and at 37 °C for 12 months, with glass as the control. They concluded that PET bottles with enhanced oxygen barrier properties better limited the formation of PV and conjugated dienes than standard PET and performed similarly to glass.

The maximum values of olive oil quality parameters for EVOO quality-grade classification are as follows [36]: free acidity, ≤0.8% oleic acid; PV, ≤20 meq O_2_/kg; K_232_, ≤2.50; K_270_, ≤0.22; ΔK, ≤0.01; Lnr = the limit value was not reached during the experimental period.

### 3.4. Evolution of Antioxidants of EVOOs and the EFSA Health Claim Under Household Use and Storage Conditions

Extensive research has indicated that during the EVOO storage, the evolution of phenolic compounds with antioxidant properties, particularly tocopherols and secoiridoid derivatives, was influenced by the level of hydrolytic and oxidative reactions, including auto-oxidation (due to the free radical triplet oxygen) and photo-oxidation (due to singlet oxygen produced in the presence of photosensitisers, such as chlorophylls) [4]. These reactions occurred concurrently and were affected by factors such as the initial composition of the EVOO [7,8,10], temperature [13,25], light exposure [9], packaging characteristics [23,27,28], and oxygen availability [14,22].

**Figure 4:**
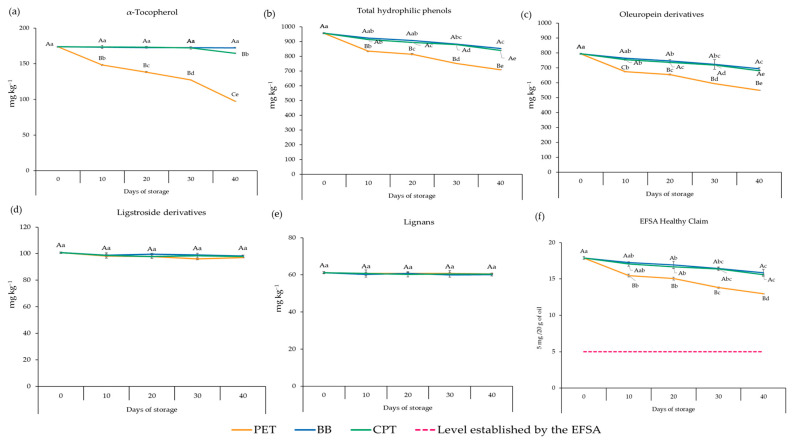
Evolution of antioxidants content (mg kg^−1^) and EFSA healthy claim in EVOOs stored within three different types of packaging under simulated household use and storage over 40 days: (**a**) α-tocopherol; (**b**) total hydrophilic phenols (expressed as the sum of oleuropein and ligstroside derivatives and lignans); (**c**) oleuropein derivatives (summed as hydroxytyrosol (3,4-DHPEA), oleacein (3,4-DHPEA-EDA), and isomer of oleuropein aglycon (3,4-DHPEA-EA)); (**d**) ligstroside derivatives (summed as tyrosol (p-HPEA), olecanthal (p-HPEA-EDA), and ligstroside aglycone); (**e**) lignans (summed as (+)-1-acetoxypinoresinol and (+)-pinoresinol); (**f**) EFSA health claim (mg hydroxytyrosol, tyrosol, and derivatives/20 g of oil) [2]. The dashed red line indicates the minimum level established by EFSA health claims related to EVOO polyphenols [2]. Results are expressed as the mean ± standard deviation of two independent determinations (*n* = 2). Uppercase letters indicate significant differences (*p* ≤ 0.05) among different EVOOs packaging at the same storage time. Lowercase letters indicate significant differences (*p* ≤ 0.05) within the same EVOO packaging during storage.

Figure 4 and Table S1 show the evolution of antioxidant levels (including α-tocopherol and hydrophilic phenolic compounds) in the EVOOs analysed in this study.

The EVOOs stored in PET experienced a higher loss of α-tocopherol, dropping from 173.8 ± 0.6 to 97.6 ± 0.2 mg kg^−1^. A significant decrease (*p* ≤ 0.05) was evident after just 10 days of simulated household use (−15%), and the reduction reached −44% after 40 days of storage. Conversely, the α-tocopherol slightly decreased in EVOOs stored in BB and CPT containers (−0.8% and −5.3%, respectively) (Figure 4a). These findings reaffirm the lower effectiveness of PET packaging compared to BB and CPT packaging in shielding EVOO under the combined effect of light and oxygen exposure. Comparable findings were reported by Sacchi et al. [23], who evaluated changes in α-tocopherol content in EVOOs packaged in different containers (PET, PET + oxygen scavenger, and glass bottles) and stored at room temperature under diffuse lighting for 6 months. A gradual decrease in α-tocopherol content in EVOOs stored under diffuse light was observed, especially in clear glass compared to PET bottles [23]. Esposto et al. [27] also found a strong correlation between light exposure and α-tocopherol depletion, reporting the best preservation of this compound when light transmission was limited. The α-tocopherol contributes to EVOO stability by donating a hydrogen atom to chain propagation, quenching singlet oxygen to decrease the level of oils’ photo-oxidation and regenerating antioxidants such as vitamin C [4,5,51]. Our findings highlight the importance of preserving α-tocopherol not just for oxidative stability but also for maintaining the bioactive profile of EVOO during storage [52].

As storage time increased, the total hydrophilic phenolic content of EVOOs significantly decreased (*p* ≤ 0.05) in all packaging containers tested in our study (Figure 4b). Notably, the EVOOs stored in PET suffered higher decreases (−26% from initial values) than those observed for EVOO stored in BB and CPT (−11% and −12%, respectively). By observing the evolution of individual fractions of hydrophilic phenols, the most evident change was recorded for oleuropein derivatives, whose concentrations lowered sharply, especially in EVOO PET stored (−31%, from 793.5 ± 3.8 to 550.4 ± 0.1 mg kg^−1^), while the EVOOs stored in BB and CPT containers showed a gradual decline (−13% and −14%, respectively), retaining significantly higher levels (693.4 ± 8.9 and 681.2 ± 1.8 mg kg^−1^, respectively) after 40 days of storage (Figure 4c). The oleacein concentration, which accounted for 61% of the initial total hydrophilic phenol content, followed a similar trend, decreasing significantly (*p* ≤ 0.05) in all EVOOs with increasing storage time (Table S1). Its content was significantly (*p* ≤ 0.05) reduced by 33%, 13%, and 11% in EVOO samples stored in PET, CPT, and BB containers, respectively. As the oleacein and isomer of the oleuropein aglycon decreased over time, hydroxytyrosol was characterised by an “up” and “down” trend, after a sudden decrease in its concentration within the range of 0 to 10 days of storage (Table S1). These results can be explained, to a certain extent, in terms of the hydrolysis of the oleacein and isomer of the oleuropein aglycon and the related release of new hydroxytyrosol [8,12]. These findings agree with those of several studies [8,12,53]. The observed trends in oleuropein derivatives can be explained by their faster capability to hamper the oxidative reactions, including both auto- and photo-oxidative processes. Their high initial concentrations provide protective effects by scavenging reactive oxygen species; however, these concentrations gradually decrease as the oxidative process continues. Esposto et al. [38] demonstrated the effectiveness of the oxidised form of oleuropein aglycon as a potential marker of the EVOO’s oxidation status. The EVOOs stored in BB and, to a lesser extent, in CPT benefited from improved light-shielding and oxygen barrier properties, which slowed degradation and better preserved oleuropein derivatives. The evolution of ligstroside derivatives was similar to that of oleuropein derivatives (Figure 4d and Table S1). However, these compounds showed slight decreases and final losses of less than 4%. At the same time, lignans remained stable, suggesting a limited role in preventing oxidation when other antioxidants were retained in EVOO [27,38,54]. The observed changes in α-tocopherol and hydrophilic phenolic compounds over 40 days of simulated household use and storage (Figure 4a–e) are consistent with the two-way ANOVA results shown in Table S2. The packaging material, storage time, and their interaction exerted a statistically significant (*p* < 0.001) effect on α-tocopherol, oleuropein derivatives, and total hydrophilic phenols. The significant interaction term indicates that the impact of storage duration on antioxidant degradation was contingent upon the packaging system, suggesting that the loss over time varied across packaging materials. Conversely, ligstroside derivatives and lignans were not significantly influenced by packaging, storage duration, or their interaction, indicating greater stability of these compounds under household storage conditions. Our findings partially support prior research indicating that Corbella EVOO stored in stainless steel containers with a nitrogen headspace and bag-in-box packaging for 12 months exhibits a significant interaction (*p* < 0.05) between storage time and packaging type for several phenolic compounds, except for lignans [33].

These results are consistent with those of Alimi et al. [14], who observed that household conditions, particularly light exposure and oxygen ingress, significantly influence phenolic retention and the maintenance of polyphenol-related health claims in Cobrançosa EVOO. Moreover, it is well known that EVOO phenols protect blood lipids from oxidative stress [14]. However, to use this health claim on labels, the concentration of these phenols, including hydroxytyrosol, tyrosol, and their complex derivatives, must exceed 250 mg kg^−1^ (equivalent to 5 mg per 20 g of olive oil) [2,3]. Even though the EVOOs analysed consistently exceeded the EU health claim standards at each sampling point, the health index of these EVOO samples experienced a significant (*p* ≤ 0.05) decline from their initial values, resulting in a loss ranging from 28% to 11% of the total phenolic content (Figure 4f and Table S1). At the end of the storage period, the lowest value was recorded for EVOO stored in PET (12.9 mg/20 g of oil), still approximately 2.6-fold above the threshold of 5 mg/20 g, while EVOO stored in BB and CPT retained 15.8 and 15.6 mg/20 g of oil, respectively, corresponding to about 3.2-fold and 3.1-fold above the threshold [2,3]. This finding aligns with that reported by Klisović et al. [34], who found that, after simulated household use for 1 month, the EVOO did not reduce its phenolic content below the levels required for the EU health claim application [2,3].

### 3.5. Evolution of Volatile Compounds of EVOOs During Household Use and Storage Conditions

**Figure 5:**
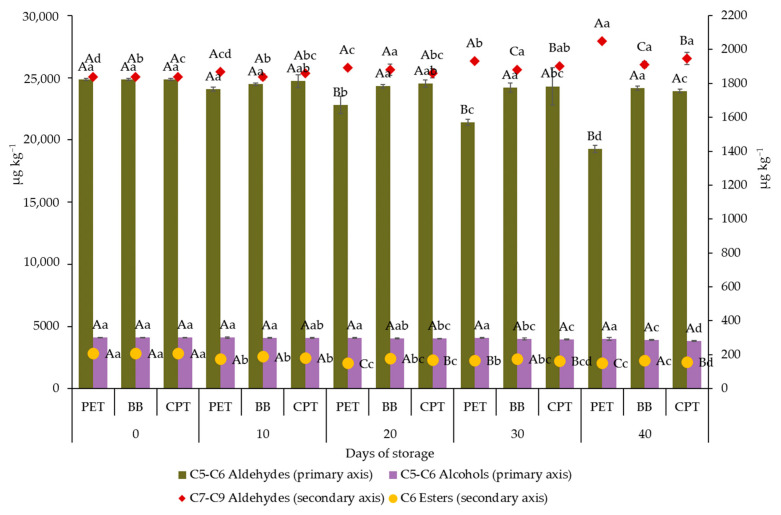
Evolution of volatile compounds (µg kg^−1^) in EVOOs stored in three different types of packaging under simulated household use and storage conditions over 40 days. Results are expressed as the mean ± standard deviation of two independent determinations (*n* = 2). Uppercase letters indicate significant differences (*p* ≤ 0.05) among different EVOOs packaging at the same storage time. Lowercase letters indicate significant differences (*p* ≤ 0.05) within the same EVOO packaging during storage. C_5_–C_6_ aldehydes (expressed as the sum of (E)-2-pentenal, (E)-2-hexenal, and (E,E)-2,4-hexadienal), C_7_–C_9_ aldehydes (the sum of heptanal, 2-heptenal, (E,E)-2,4-heptadienal, and nonanal), C_5_–C_6_ Alcohols (the sum of 1-pentanol, 1-penten-3-ol, (E)-2-penten-1-ol, (Z)-2-penten-1-ol, 1-hexanol, (E)-2-hexen-1-ol, and (Z)-3-hexen-1-ol), C_6_ Esters (the sum of hexyl acetate and (Z)-3-hexenyl acetate).

**Figure 6:**
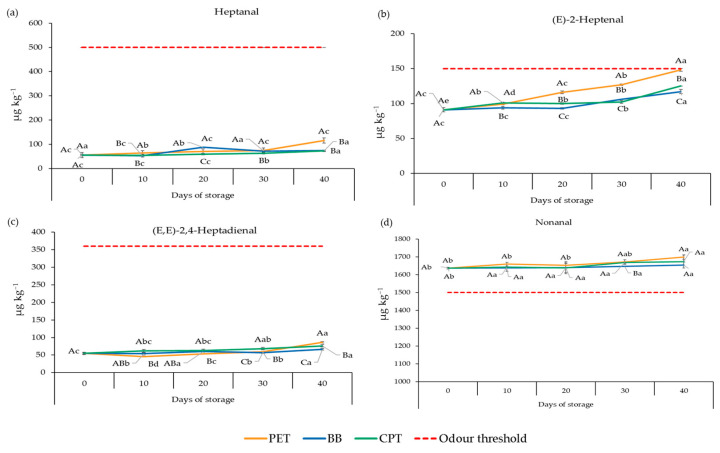
Evolution of heptanal (**a**), (E)-2-heptenal (**b**), (E)-2,4-heptadienal (**c**), and nonanal (**d**) (µg kg^−1^) in EVOOs stored in three different types of packaging under simulated household storage and use conditions over 40 days. The dashed red line indicates the odour perception threshold: 500 µg kg^−1^ for heptanal [58], 150 µg kg^−1^ for (E)-2-heptenal [48], 363 µg kg^−1^ for (E,E)-2,4-heptadienal [58], and 1500 µg kg^−1^ for nonanal [58]. Results are expressed as the mean ± standard deviation of two independent determinations (*n* = 2). Uppercase letters indicate significant differences (*p* ≤ 0.05) among different EVOOs packaging at the same storage time. Lowercase letters indicate significant differences (*p* ≤ 0.05) within the same EVOO packaging during storage.

Various classes of volatile compounds were identified in the headspace of EVOO, including aldehydes, alcohols, esters, ketones, hydrocarbons, terpenes, and carboxylic acids. Most of these compounds, particularly those derived from the lipoxygenase (LOX) pathway, are key contributors to its distinctive flavour [42,44,46,55,56,57,58,59], while others are associated with sensory defects [43,47,49,57] (Table S3). The evolution of volatile compounds, which primarily affects the sensory qualities of EVOOs stored under common household conditions (i.e., exposure to light and oxygen), is illustrated in Figure 5 and Figure 6. All quantitative data on the identified volatile compounds are presented in Table S4.

By simulating home storage and use, significant changes (*p* ≤ 0.05) in the volatile profiles of EVOOs were observed across all three packaging types (Table S4). At the initial time point, (E)-2-hexenal was the most dominant volatile compound in EVOOs, followed by (Z)-3-hexen-1-ol, (E)-2-hexen-1-ol, and 1-penten-3-one (Table 1 and Table S4). These volatile compounds mainly contribute to green and fruity sensory notes [44,54,56] (Table S3). Overall, the LOX-derived compounds, including saturated and unsaturated C_5_–C_6_ aldehydes, C_5_–C_6_ alcohols, and C_6_ esters, showed a significant decrease (*p* ≤ 0.05) after 40 days of storage (Figure 5). In contrast, the C_7_–C_9_ aldehydes, which several authors have previously associated with rancidity [48,51,55], accumulated significantly (*p* ≤ 0.05) (Table S4). After 40 days, the greatest reduction in C_5_–C_6_ aldehydes synthesised through the LOX pathway from linoleic and linolenic acids was observed in EVOO stored in PET containers, with a 22.5% decrease. In comparison, EVOOs stored in BB and CPT retained more of these compounds, with reductions of only 2.7% and 3.7%, respectively. Notably, the content of (E)-2-hexanal significantly decreased (*p* ≤ 0.05) over time and was found to be significantly (*p* ≤ 0.05) higher in EVOOs stored in PET than in those stored in BB and CPT (Table S4). Its concentration dropped from an initial value of 24,697 ± 73 μg kg^−1^ across the three packaging types tested, resulting in reductions of 23%, 4%, and 3% for EVOOs stored in PET, CPT, and BB, respectively, indicating a loss of freshness [42,44,57]. In line with previous studies, Kalua et al. [60] reported a progressive decline in herbaceous and fruity olfactory notes during storage, which was positively correlated with decreasing levels of (E)-2-hexenal. This compound has been identified as a key quality marker for EVOO stored in the dark. Also, the (E)-2-pentenal and (E,E)-2,4-hexadienal followed the same trend as (E)-2-hexanal, with a more pronounced decrease in EVOOs stored in PET than in those stored in BB and CPT (Table S4).

In our study, several other volatile compounds belonging to the chemical classes of alcohols and esters such as 1-penten-3-ol, (E)-2-penten-1-ol, (Z)-3-hexenyl acetate, and hexyl acetate, significantly (*p* ≤ 0.05) decreased within the initial 40 days of storage (Table S4). This decline was more pronounced in EVOOs stored in PET containers than in those stored in CPT and BB. The literature indicates that plastic packaging materials, including PET, may absorb volatile aroma and flavour compounds from EVOO, thereby potentially affecting its sensory characteristics [61]. Although this study did not directly evaluate volatile absorption by the PET, BB, and CPT, it is plausible that the observed reduction in low-molecular-weight volatile compounds may be partly attributable to a possible scalping effect associated with plastic packaging materials [15,29,61]. A similar phenomenon may also occur in bag-in-box packaging; however, because the flexible inner bag contains less plastic material and has a smaller contact area with EVOO than conventional PET containers, this effect is likely to be less pronounced.

The effectiveness of different packaging materials was further assessed by monitoring the levels of C_7_–C_9_ aldehydes, which are widely used as markers of oxidative rancidity in EVOOs exposed to light and oxygen [10,43,48]. Notably, heptanal and nonanal are derived from the decomposition of hydroperoxides associated with the autoxidation of oleic acid, whereas (E)-2-heptenal results from secondary oxidation reactions of linoleic acid [56]. As shown in Table S4, a significant increase (*p* ≤ 0.05) in the concentration of C_7_–C_9_ aldehydes, including heptanal, (E)-2-heptenal, (E,E)-2,4-heptadienal, and nonanal, was observed as a function of storage time and packaging type. From days 0 to 40, EVOOs stored in PET showed the highest relative increase in C_7_–C_9_ aldehydes, with heptanal, (E)-2-heptenal, and (E,E)-2,4-heptadienal increasing by approximately 107%, 63%, and 56%, respectively (Figure 6). Nevertheless, the final concentrations of these compounds (116, 148, and 86 µg kg^−1^, respectively) remained below their reported odour perception thresholds [43,56,58], although (E)-2-heptenal approached its threshold value at the end of storage (Figure 6b). In EVOOs stored in BB and CPT, the relative increases in the same aldehydes were markedly lower, ranging from approximately 20% to 38%, confirming the superior protective effect of these packaging materials against oxidative degradation. In these samples, C_7_ aldehyde concentrations, including heptanal, (E)-2-heptenal, and (E,E)-2,4-heptadienal, remained well below their respective sensory thresholds over time [43,56,58]. The nonanal exhibited only minor relative changes during storage, with increases of approximately 1–4% across packaging materials. Although nonanal was present at relatively high absolute concentrations (up to ~1700 µg kg^−1^), its reported odour threshold in EVOO is considerably higher (approximately 1000–2000 µg kg^−1^), and its limited relative increase suggests a restrained contribution to rancid sensory notes under the investigated storage conditions [58]. Therefore, although its concentration exceeds this threshold range, the limited relative increase can indicate only a minor contribution to rancid sensory notes under the storage conditions studied. Overall, these results align with the sensory findings, showing that the rise in oxidation-related aldehydes did not lead to a detectable rancid defect, as their concentrations mostly stayed below, or only slightly near, the reported odour perception thresholds, except for nonanal [59]. In addition, the 6-methylhept-5-en-2-one, associated with rancidity defects detected in EVOO [58], exhibited a significant (*p* ≤ 0.05) increase exclusively in EVOOs stored in PET, while its concentration decreased in samples stored in BB and CPT (Table S4). Over 40 days, the EVOOs stored in PET showed the most pronounced increase in volatile compounds of oxidative origin, due to packaging materials with high oxygen permeability [29]. These findings are consistent with those of Cecchi et al. [43], who identified light exposure as the primary factor driving the formation of oxidation-related volatile compounds from both polyunsaturated and monounsaturated fatty acids. Overall, in agreement with the literature, BB proved to be the most effective packaging material for EVOO storage, as it better preserved LOX-derived volatile compounds and limits the accumulation of secondary oxidation products associated with rancidity, unlike PET.

### 3.6. Evolution of Sensory Profile of EVOOs During Household Use and Storage Conditions

**Figure 7:**
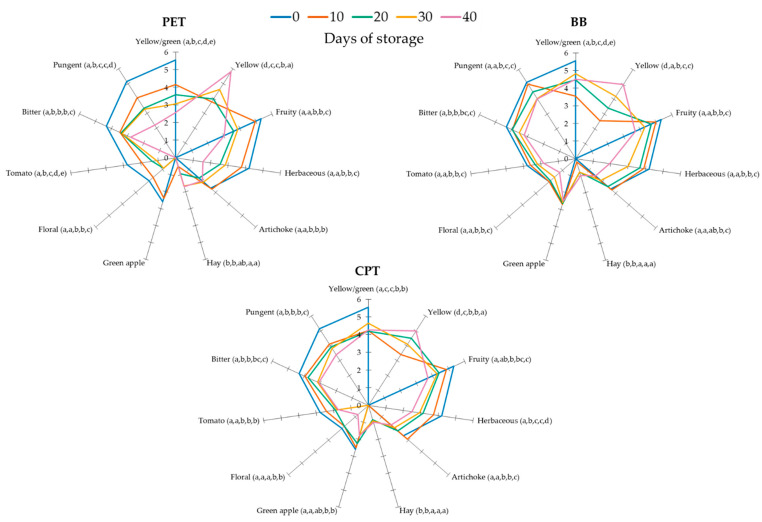
Evolution of the sensory profile of EVOOs stored in different packaging materials, PET, BB, and CPT, respectively, under household storage and use conditions. Results are the mean of the sensory evaluation performed by 10 experienced panellists. Different letters in brackets indicate significant differences among storage times (0–40 days) for the same sensory attribute (*p* < 0.05).

As established by UE regulation [36], EVOO is the only food product whose commercial classification (extra virgin, virgin, or lampante olive oil) is legally defined and based on sensory analysis; hence, the role of packaging material in EVOOs’ sensory quality was investigated under household use and storage conditions. Figure 7 illustrates the evolution of EVOO profiles for three different packaging types (PET, BB, and CPT). After 40 days of opening, all samples remained classified as EVOO (defect median = 0; fruity median > 0) [36,41]. As discussed earlier, at the initial storage time, the EVOO samples displayed strong positive sensory attributes associated with green and fruity sensory notes from various volatile compounds originating from the LOX pathway (C_5_-C_6_ saturated and unsaturated aldehydes, the related alcohols and esters), typically including herbaceous, tomato, artichoke, and pronounced bitter and pungent gustatory–retronasal attributes [44]. During the SSL, all analysed EVOO samples showed a significant (*p* < 0.05) reduction in olfactory attributes, such as fruity, herbaceous, green apple, artichoke, and tomato, as well as bitterness and pungency [45]. Meanwhile, yellow and hay notes increased when exposed to light and oxygen, conditions common in EVOO stored at home. However, EVOOs stored in BB and CPT showed higher intensities of fruity, herbaceous, artichoke, apple, and tomato attributes than those stored in PET, and this occurred at all time points examined. In contrast to previous studies [33,43] that reported rancidity of EVOO after approximately 3 weeks stored under simulated home-use conditions (under light exposure in the presence of oxygen), in this study, none of the EVOO samples developed rancid defects. This result can be partly explained by the initially high intensity of fruity and green attributes due to the high concentration of LOX-derived volatile compounds, which may partially mask rancid off-flavour [44]. Furthermore, although some volatile compounds related to oxidation increased during storage, their levels were mostly below odour thresholds. Heptanal and (E,E)-2,4-heptadienal stayed well below sensory thresholds, while (E)-2-heptenal neared its threshold only at the last time point in EVOO stored in PET. Nonanal, despite being above its odour threshold, showed minor changes. Thus, the increase in oxidation markers could not induce a perceptible rancid defect. These observations align with previous studies’ findings regarding the intensity of some sensory notes, which can in fact be masked or enhanced by others, particularly in VOOs rich in phenolic compounds [44,45]. Additionally, as reported by Genovese et al. [44], EVOOs with high phenolic content (above 500 mg kg^−1^) are more resistant to the development of minor sensory defects during storage, thus preserving the “extra virgin” classification for longer. In this study, even EVOOs stored in PET retained relatively high levels of total phenols at the end of the storage period (approximately 700 mg kg^−1^), which likely contributed to limiting the onset of sensory defects.

Overall, the results confirm that packaging plays a key role in preserving EVOO sensory quality during household storage and use, with BB and CPT systems better maintaining the positive sensory attributes compared to PET packaging. After 40 days of storage, the EVOOs packaged in PET suffered a qualitative decline, detectable primarily through the reduction in tomato attributes, and the complete disappearance of green apple on day 20, while bitter and pungent attributes persisted. Excessive oxygen exposure, due to the headspace created in the container after each sampling, combined with the PET bottles’ permeability to oxygen and light, led to more rapid deterioration in oil quality for EVOO samples. Similar results were reported by Pristouri et al. [16] and Sacchi et al. [23]. For both the EVOOs stored in BB and CPT, the panel test revealed media with fruity, herbaceous, bitter, and pungent notes higher than those stored in PET, reflecting their higher concentrations of phenolic and volatile compounds. This is not surprising, and similar behaviour was already reported in a previous study by Lolis et al. [25], which investigated the effect of BB as a packaging material on the preservation of the qualitative characteristics of EVOO stored under home conditions.

## 4. Conclusions

This study examined how the regulatory, sensory, and health-related qualities of EVOO change when stored in three different packaging materials—PET, BB, and CPT—over 40 days of simulated household use. Our findings underscore the need to jointly consider packaging type and storage conditions when evaluating oil stability.

The PET container exhibited the least protection, with peroxide values exceeding the legal limit for the “extra virgin” category after about 30–40 days, declassifying the sample “lampante”. Additionally, EVOO in PET experienced significant decreases in α-tocopherol and oleuropein derivatives, along with a notable drop in LOX-derived volatiles linked to positive sensory qualities, compared to oils in BB and CPT. Overall, this study provides useful information on the performance of commercially available packaging systems under conditions simulating household use, including repeated opening, headspace formation, oxygen exposure, and light exposure. The results emphasise the importance of packaging materials with low oxygen permeability and effective light barrier properties, especially for short- to medium-term storage after opening for preserving EVOO quality. While the PET tested in our study is popular due to its convenience and low cost, the findings suggest it is not the best option for preserving EVOO during household use. The BB container was the most effective among those tested, providing better protection against light and oxygen in real-world use. The relatively elevated initial phenolic content of the EVOO analysed in this study may have contributed to its inherent oxidative stability. The results obtained, therefore, refer to the specific EVOO and experimental conditions considered, and different oils may show different stability during storage. Accordingly, conducting further research on EVOOs with diverse phenolic and antioxidant profiles would help expand the applicability of these results.

From a practical perspective, when EVOO is intended for consumption within 1–2 months after opening, packaging systems such as bag-in-box followed by tin-plated cans should be preferred over PET containers to better preserve oil quality.

## Figures and Tables

**Table 1:** Initial chemical composition and sensory profile of the EVOO used in this study (T0).

Parameters	EVOO
Chemical composition	
Free acidity (%)	0.28 ± 0.01
Peroxide value (meq O_2_/kg)	4.8 ± 0.001
K_232_	1.65 ± 0.05
K_270_	0.11 ± 0.01
ΔK	0.0015 ± 0.0001
α-Tocopherol (mg kg^−1^)	173.8 ± 0.6
Total hydrophilic phenols (mg kg^−1^)	956 ± 4
Oleuropein derivatives (mg kg^−1^)	793.55 ± 3.81
Ligstroside derivatives (mg kg^−1^)	100.67 ± 0.44
Lignans (mg kg^−1^)	61.2 ± 0.2
C_5_–C_6_ Aldehydes (µg kg^−1^)	24,860.1 ± 73.1
C_7_–C_9_ Aldehydes (µg kg^−1^)	1838.7 ± 6.4
C_5_–C_6_ Alcohols (µg kg^−1^)	4116 ± 9.3
C_6_ Esters (µg kg^−1^)	209.9 ± 0.9
C_5_–C_7_ Ketones (µg kg^−1^)	1152.88 ± 16.1
Sensory attributes	
Yellow/green	5.54 ± 0.14
Fruity	5.33 ± 0.92
Herbaceous	4.20 ± 0.10
Artichoke	1.13 ± 0.20
Green apple	0.37 ± 0.06
Floral (esters)	0.29 ± 0.05
Tomato	1.59 ± 0.30
Bitter	4.31 ± 0.10
Pungent	5.14 ± 0.11
Off-flavours	n.p.

Chemical data are the mean of two independent determinations ± standard deviation. Sensory data are the mean of the sensory evaluation performed by 10 trained panellists. Total hydrophilic phenols (expressed as the sum of oleuropein and ligstroside derivatives and lignans), oleuropein derivatives (the sum of hydroxytyrosol (3,4-DHPEA), isomer of the oleuropein aglycon (3,4-DHPEA-EA), and oleacein (3,4-DHPEA-EDA)), ligstroside derivatives (the sum of tyrosol (p-HPEA) and oleocanthal (p-HPEA-EDA)), lignans (the sum of (+)-1-acetoxypinoresinol and (+)-pinoresinol), C_5_–C_6_ Aldehydes (the sum of (E)-2-pentenal, (E)-2-hexenal, and (E,E)-2,4-hexadienal), C_7_–C_9_ Aldehydes (the sum of heptanal, (E)-2-heptenal, (E,E)-2,4-heptadienal, and nonanal), C_5_–C_6_ Alcohols (the sum of 1-pentanol, 1-penten-3-ol, (E)-2-penten-1-ol, (Z)-2-penten-1-ol, 1-hexanol, (E)-2-hexen-1-ol, and (Z)-3-hexen-1-ol) C_6_ Esters (the sum of hexyl acetate and (Z)-3-hexenyl acetate), C_5_–C_8_ Ketones (the sum of 3-pentanone, 1-penten-3-one, and 6-methyl-5-hepten-2-one), n.p., not perceived.

**Table 2:** Changes in regulatory quality parameters of EVOO under simulated household storage and use.

Parameter	EVOO	R^2^	Storage Time (Days) to Exceed the Regulatory Quality Limit	Increase Rate
			(range of 10 days)	
Free acidity	PET	0.45	Lnr	0.00030
	BB	0.89	Lnr	0.00050
	CPT	0.90	Lnr	0.00060
PV	PET	0.90	30–40 days	0.54700
	BB	0.61	Lnr	0.00900
	CPT	0.03	Lnr	0.00400
K_232_	PET	0.98	Lnr	0.00668
	BB	0.91	Lnr	0.00068
	CPT	0.67	Lnr	0.00133
K_270_	PET	0.74	Lnr	0.00077
	BB	0.95	Lnr	0.00003
	CPT	0.94	Lnr	0.00040
ΔK	PET	0.58	Lnr	0.00050
	BB	0.49	Lnr	0.00041
	CPT	0.49	Lnr	0.00041

## Data Availability

The original contributions presented in this study are included in the article/Supplementary Material. Further inquiries can be directed to the corresponding author.

## Supplementary Materials

The following supporting information can be downloaded at: https://www.mdpi.com/article/10.3390/foods15111948/s1, Table S1: Evolution of concentration of α-tocopherol and hydrophilic phenolic compounds (mg kg^−1^) and the EFSA health claim in EVOOs stored within three different types of packaging under simulated household storage and use over 40 days; Table S2: Results of two-way ANOVA evaluating the effects of packaging material and storage time, and their interaction, on α-tocopherol and phenolic compounds under household use and storage conditions; Table S3: Volatile compounds, chemical structures, sensory attributes, and odour thresholds (μg kg^−1^ oil) in EVOO. Adapted from [44,48,55,56,57,58,59]; Table S4: Evolution of volatile compounds (µg kg^−1^) in EVOOs stored in three different types of packaging under simulated household storage and use over 40 days. Figure S1. EVOO packaging materials: (a) green food-grade polyethylene terephthalate (PET) container (PET packaging); (b) bag-in-box (BB) system consisting of a multilayer pouch placed inside a parallelepiped-shaped corrugated paperboard box (BB packaging); and (c) tin-plated parallelepiped can with an internal varnish based on chrome-plated stripe coating (CPT packaging). Reference [62] is cited in the supplementary materials.
