# Supplementary material for: Evaluating the Effect of Packaging Materials on Extra Virgin Olive Oil Quality Under Simulated Household Use and Storage Conditions
Source: Foods. 2026 Jun 1;15(11):1948. doi: 10.3390/foods15111948 (PMC13257077; doi:10.3390/foods15111948)
Supplement: Supplementary file 1 [file foods-15-01948-s001.zip › foods-4211539-supplementary.pdf]

Table S1. Evolution of concentration of α-tocopherol and hydrophilic phenolic compounds (mg kg^{-1}) and the EFSA Health Claim in EVOOs stored within three different types of packaging under simulated household storage and use over 40 days.

	Days of storage				
	0	10	20	30	40
			PET		
α-Tocopherol	173.8±0.6Aa	148.2±0.9Bb	138.2±1Bc	127.2±0.4Bd	97.6±0.2Ce
Hydroxytyrosol (3,4-DHPEA)	11.5±0.01Aa	5.0±0.1Be	5.3±0.01Ad	5.8±0.01Ab	5.6±0.01Ac
Tyrosol (p-HPEA)	4.0±0.1Aa	2.8±0.2Ad	3.0±0.1ABc	3.1±0Abc	3.3±0.01Ab
Vanillic acid	0.3±0.01Aa	0.3±0.01Aa	0.3±0.01Aa	0.3±0.01Aa	0.3±0.01Aa
p-Cumaric acid	0.3±0.01Aa	0.2±0.01Aa	0.2±0.01Aa	0.2±0.01Aa	0.2±0.01Aa
Oleacein (3,4-DHPEA-EDA)	586.1±3.8Aa	481.7±0.7Cb	474.3±2.9Bc	428.3±0.8Bd	394.3±0Be
Oleocanthal (p-HPEA-EDA)	77.8±0.4Aa	77.1±0.3Aab	76.5±0.3Abc	75.6±0.1Ad	76.5±0.1Ac
(+)-1-Acetoxypinoresinol	39.2±0.01Aa	39.0±0.01Ab	39.0±0.01A	38.9±0.01Ac	39.0±0.01Ab
(+)-Pinoresinol	22.0±0.2Aa	21.8±0.01Aa	21.8±0.6Aa	21.8±0.2Aa	21.6±0.7Aa
Isomer of the oleuropein aglycon (3,4-DHPEA-EA)	195.9±0.5Aa	188.1±2.5Ab	175.3±2.6Ac	160±0.1Ad	150.5±0.1Be
Ligstroside aglicone	18.8±0.1Aa	18.3±0.4Aa	18.3±0.2Ba	17.4±0.7Bb	17.2±0.1Bb
Oleuropein derivatives	793.5±3.8Aa	674.8±2.6Cb	654.9±3.9Bc	594.1±0.8Bd	550.4±0.1Be
Ligstroside derivatives	100.7±0.4Aa	98.2±0.6Aab	97.8±0.4Ab	96.1±0.7Ab	97±0.2Ab
Lignans	61.2±0.2Aa	60.8±0.01Aa	60.8±0.6Aa	60.7±0.2Aa	60.5±0.7Aa
Total hydrophilic phenols	956.0±3.9Aa	834.2±3.6Bb	814.0±4.7Bc	751.3±1.1Bd	708.4±0.7Be
EFSA health claim [2]	17.9±0.2Aa	15.5±0.2Bb	15.1±0.2Bb	13.8±0.1Bc	12.9±0.001Bd
			BB		
α-Tocopherol	173.8±0.6Aa	173.1±0.7Aa	172.5±0.6Aa	172.4±0.1Aa	172.3±0.7Aa
Hydroxytyrosol (3,4-DHPEA)	11.5±0.01Aa	3.9±0.01Cb	3.8±0.1Cb	4.5±0.1Bc	4.8±0.01Cd
Tyrosol (p-HPEA)	4.0±0.1Aa	2.7±0.01Ac	3.5±0.5Ab	3±0.1Abc	3.0±0.01Bbc
Vanillic acid	0.3±0.01Aa	0.3±0.01Aa	0.3±0.01Aa	0.3±0.01Aa	0.3±0.01Aa
p-Cumaric acid	0.3±0.01Aa	0.3±0.01Aa	0.2±0.01Aa	0.2±0.01Aa	0.2±0.01Aa
Oleacein (3,4-DHPEA-EDA)	586.1±3.8Aa	575.5±0.8Abc	561.1±8.6Ac	544.8±12.2A	519.5±8Ae
Oleocanthal (p-HPEA-EDA)	77.8±0.4Aa	77.4±2.4Aa	77.3±0.5Aa	77.3±4.2Aa	76.9±1.7Aa
(+)-1-Acetoxypinoresinol	39.2±0.01Aa	39.0±0.1Ab	38.8±0.01Cc	38.8±0.01Bc	38.8±0.01Bc
(+)-Pinoresinol	22.0±0.2Aa	21.3±0.6Aa	21.9±0.3Aa	21.2±1.01Aa	21.2±0.3Aa
Isomer of the oleuropein aglycon (3,4-DHPEA-EA)	195.9±0.5Aa	185.0±0.7Aa	181.2±5Aa	173.6±30.3Aa	169.1±3.9Aa
Ligstroside aglicone	18.8±0.1Aa	18.7±0.6Aa	18.8±0.2Aa	18.8±0.1Aa	18.3±0.3Aa
Oleuropein derivatives	793.5±3.8Aa	764.5±1Aab	746.2±10Ab	723±32.7Abc	693.4±8.9Ac
Ligstroside derivatives	100.7±0.4Aa	98.9±2.5Aa	99.6±0.8Aa	99.1±4.2Aa	98.2±1.7Aa
Lignans	61.2±0.2Aa	60.3±0.6Aa	60.7±0.3Aa	60.0±1.01Aa	60.1±0.3Aa
Total hydrophilic phenols	956.0±3.9Aa	924.2±2.8Aab	907.1±11.2A	882.7±44.8A	852.3±9.8Ac
EFSA health claim [2]	17.9±0.2Aa	17.3±0.1Aab	16.9±0.5Ab	16.4±0.2Abc	15.9±0.5Ac
			CPT		
α-Tocopherol	173.8±0.6Aa	173.5±2Aa	173.1±1.4Aa	172.3±1.8Aa	164.6±0.5Bb
Hydroxytyrosol (3,4-DHPEA)	11.5±0Aa	5.8±0.2Ab	4.9±0Bc	5.9±0.1Ab	5.1±0.1Bc
Tyrosol (p-HPEA)	4.0±0.1Aa	2.7±0Ac	2.7±0Bc	3.1±0.1Ab	3.0±0.1Bb
Vanillic acid	0.3±0.01Aa	0.3±0.01Aa	0.3±0.01Aa	0.3±0.01Aa	0.3±0.01Aa
p-cumaric acid	0.3±0.01Aa	0.2±0.01Aa	0.2±0.01Aa	0.2±0.01Aa	0.2±0.01Aa
Oleacein (3,4-DHPEA-EDA)	586.1±3.8Aa	564.1±2.7Bb	547.9±1.3Ac	535.8±2.5Ad	510.2±1.8Ae
Oleocanthal (p-HPEA-EDA)	77.8±0.4Aa	77.1±0.1Aa	77±1.2Aa	77.1±1.4Aa	77.1±0.2Aa
(+)-1-Acetoxypinoresinol	39.2±0.01Aa	39.0±0.01Ab	38.9±0.01Bb	38.9±0.01Ab	38.8±0.1Bb
(+)-Pinoresinol	22±0.2Aa	21.7±0.2Aa	21.4±0.4Aa	21.6±0.6Aa	21.5±1.4Aa
Isomer of the oleuropein aglycon (3,4-DHPEA-EA)	195.9±0.5Aa	184.4±3.6Ab	183.2±3.1A	177.7±1.2Ac	165.8±0.4Ad
Ligstroside aglicone	18.8±0.1Aa	18.8±0.01Aa	18.1±0.01Bb	18.2±0.1Ab	17.7±0.3Bc
Oleuropein derivatives	793.5±3.8Aa	754.3±4.5Bb	736.0±3.4Ac	719.3±2.8Ad	681.2±1.8Ae
Ligstroside derivatives	100.7±0.4Aa	98.6±0.2Aab	97.8±1.2Ab	98.4±1.4Ab	97.8±0.3Ab
Lignans	61.2±0.2Aa	60.7±0.2Aa	60.3±0.4Aa	60.5±0.6Aa	60.4±1.4Aa
Total hydrophilic phenols	956.0±3.9Aa	914.1±5.7Ab	894.6±4.7Ac	878.7±3.4Ad	839.9±2.4Ae
EFSA health claim [2]	17.9±0.2Aa	17.1±0.3Aab	16.7±0.2Ab	16.4±0.2Abc	15.6±0.1Ac

Results are the mean of two independent determinations ± the standard deviation. Different uppercase letters (A-C) in the column indicate statistically significant ($p \leq 0.05$) differences among different EVOOs packaging at the same storage time. Different lowercase letters (a-e) in the row indicate statistically significant ($p \leq 0.05$)

differences within the same EVOO packaging during storage. Total hydrophilic phenols (expressed as the sum of oleuropein and ligstroside derivatives and lignans); Oleuropein derivatives (summed as hydroxytyrosol (3,4-DHPEA), oleacein (3,4-DHPEA-EDA), and isomer of oleuropein aglycon (3,4-DHPEA-EA)); Ligstroside derivatives (summed as tyrosol (p-HPEA), olecanthal (p-HPEA-EDA), and ligstroside aglycone); Lignans (summed as (+)-1-acetoxypinoresinol and (+)-pinoresinol); EFSA health claim (mg hydroxytyrosol, tyrosol, and derivatives/20 g of oil) [2].

Table S2. Results of two-way ANOVA evaluating the effects of packaging material and storage time, and their interaction, on α-tocopherol and phenolic compounds under household use and storage conditions.

Compound	Packaging	Days	Interaction
α-Tocopherol	$p < 0.001$	$p < 0.001$	$p < 0.001$
Oleuropein derivatives	$p < 0.001$	$p < 0.001$	$p < 0.001$
Ligstroside derivatives	ns	ns	ns
Lignans	ns	ns	ns
Total hydrophilic phenols	$p < 0.001$	$p < 0.001$	$p < 0.001$

Data were analysed by two-way ANOVA considering packaging material and storage time as fixed factors, including their interaction. When significant effects were observed, Tukey's post hoc test was applied. Statistical significance was set at $p < 0.001$; $p < 0.05$; ns = not significant.

Table S3. Volatile compounds, chemical structures, sensory attributes, and odour thresholds (µg kg^{-1} oil) in EVOO. Adapted from [44,48,55,56,57,58,59].

Compounds	Chemical structure	Sensory attributes	Odour threshold (µg kg^{-1} oil)	Ref
		Aldehydes		
Propanal	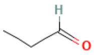	Pungent, sweet, floral, fresh, fruit, malt	9.4	[55]
Pentanal	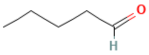	Woody, bitter, oily	240	[58]
(E)-2-Pentenal	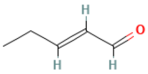	Green, apple	300	[58]
Hexanal	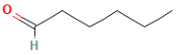	Green apple, grassy	80	[58]
(E)-2-Hexenal	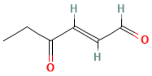	Green, apple-like	424	[56]
(E,E)-2,4-Hexadienal	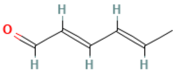	Green, floral, cut grass, fresh, fat, solvent, citric	2000	[55]
Heptanal	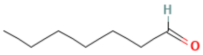	Oily, fatty, woody	500	[58]
(E)-2-Heptenal	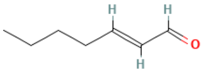	Soap, fat, bitter almond, green, greasy, pungent, oxidized, wood, tallow, grass	5, 150	[48,57,58]
(E,E)-2,4-Heptadienal	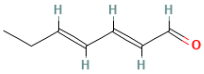	Nut, fat, rancid, oil, green	360, 710, 3620, 10000	[55,56,57,58]
Nonanal	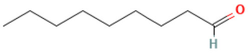	Fat, citrus, grass, green, rancid, wax, pungent, soap, tallow	1500	[58]
		Alcohols		
1-Pentanol	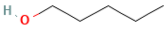	Balsamic, fruity, pungent, spicy, sticky, sweet, strong	470, 3000	[55,58]
1-Penten-3-ol	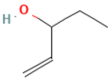	Butter, green, fresh, grass, banana, fruity, nut, wet earth, lawn, hay, pungent, olive leaf	400	[58]
(E)-2-Penten-1-ol	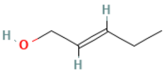	Grass, green, perfumery, wood	400	[58]
(Z)-2-Penten-1-ol	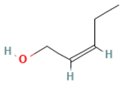	Green, plastic, fat, butter, rubber, pungent, almond, banana, olive, fruity, grass, sweet, walnut husk	250	[55,56]
1-Hexanol	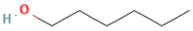	Resin, floral, green, grass, fruit, aromatic, banana, alcoholic, rough, astringent, soft, sweet, tomato	400	[55,56]
(E)-2-Hexen-1-ol	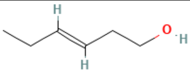	Green grass, leaves green, fruity, sweet, bitter, astringent, lawn, fat, pungent, walnut, apple, flora	5000, 8000	[55,56,57,58]
(Z)-3-Hexen-1-ol	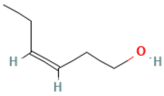	Olive paste, green, leaf, grass, herb, apple, banana, pungent, fresh, fruit, sweet	1100, 1500, 6000	[55,56,57,58]
		Esters		
Hexyl acetate	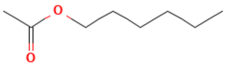	Green, fruity, sweet	1040	[55]

(Z)-3-Hexenyl Acetate	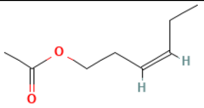	Banana, fruity, green, floral, leaves, olives, swee	200, 750	[55,56]
		Ketones		
3-Pentanone	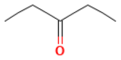	Ether, sweet, fruit, green, bitter, mustard	70000	[56]
1-Penten-3-one	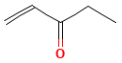	Green, fish, metallic, pungent, train oil, sharp, mustard, spicy, strawberry, leaf, bitter, sweet	0.73, 50	[55,56,58]
6-Methyl-5-hepten-2-one	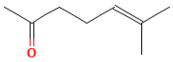	Mushroom, rubber, pepper, green, grass, pungent, banana, fruity, herb	1000	[55]

Chemical structure of each volatile compound was from National Center for Biotechnology Information [62].

Table S4. Evolution of volatile compounds (μg kg^{-1}) in EVOOs stored in three different types of packaging under simulated household storage and use over 40 days.

	Days of storage				
	0	10	20	30	40
			PET		
Aldehydes					
Propanal	59±1Ac	62±2Ac	62±5Ac	70±3Ab	85±1Aa
Pentanal	14±1Ac	19±2Abc	20±4Aab	22±1Aab	25±1Aa
(E)-2-Pentenal	67±0Aa	62±1Bb	61±2Bb	56±1Bc	54±3Bc
Hexanal	525±6Ad	682±18Ac	793±20Ab	745±23Ab	819±45Aa
(E)-2-Hexenal	24697±73Aa	23933±158A	22670±688B	21281±236Bc	19136±295Bd
(E,E)-2,4-Hexadienal	96±0Aa	96±2Aa	89±2Ab	83±2Bc	82±2Ac
Sum of C_5-C_6 aldehydes	24860±73Aa	24091±158A	22820±688B	21420±236Bc	19273±295Bd
Heptanal	56±2Ac	64±3Ac	71±1Bbc	75±3Ab	116±6Aa
(E)-2-Heptenal	91±3Ae	99±1Ad	116±2Ac	127±1Ab	148±2Aa
(E,E)-2,4-Heptadienal	55±1Ac	46±2Bd	53±0Bc	59±1Bb	86±2Aa
Nonanal	1636±5Ab	1659±10Ab	1653±13Ab	1671±13Aab	1699±12Aa
Sum of C_7 - C_9 aldehydes	1839±6Ad	1868±11Acd	1892±13Ac	1931±13Ab	2049±14Aa
Alcohols					
1-Pentanol	41±1Aa	42±1Aa	41±1Aa	40±0Aa	45±17Aa
1-Penten-3-ol	268±0Aa	261±1Aa	260±3Aa	264±4Aa	240±8Ab
(E)-2-Penten-1-ol	43±1Aa	42±1Aa	42±1Aa	40±2Aab	37±1ABb
(Z)-2-Penten-1-ol	360±2Aa	355±11Aa	354±11Aa	355±9Aa	350±5Aa
1-Hexanol	606±8Aa	603±2Aa	602±21Aa	596±7Aab	567±10Ab
(E)-2-Hexen-1-ol	1174±4Aa	1172±34Aa	1170±40Aa	1169±4Ab	1168±110Ac
(Z)-3-Hexen-1-ol	1625±3Aa	1623±35Aa	1616±17Aa	1605±26Aa	1595±30Aa
Sum of C_5-C_6 alcohols	4116±9Aa	4098±50Aa	4084±50Aa	4069±29Aa	4003±116Aa
Esters					
Hexyl acetate	21±1Aa	19±1Aab	17±1Abc	17±1Abc	14±1ABc
(Z)-3-Hexenyl Acetate	189±0Aa	155±9Ab	133±1Cd	149±4Bbc	137±7Acd
Sum of C_6 esters	210±1Aa	174±9Ab	150±11Bc	167±4Bb	151±7Cc
Ketones					
3-Pentanone	237±7Aa	224±0Aa	221±13Ba	225±2Ba	219±11Ba
1-Penten-3-one	892±14Aa	886±8Aab	882±2Aab	856±16Aab	853±20Ab
6-Methyl-5-hepten-2-one	23±0Aa	19±0Ba	22±0Aa	24±1Aa	52±4Ab
Sum of C_5 - C_8 ketones	1153±16Aa	1129±8Ba	1125±13Aa	1105±16Aa	1124±23Aa
			BB		
Aldehydes					
Propanal*	59±1Ac	58±1Ab	61±2Ab	66±2Aa	69±3Ba
Pentanal	14±1Ac	13±1Bb	20±0Aa	21±0Aa	20±2Ba
(E)-2-Pentenal	67±0Aa	64±0Ba	67±0Aa	67±1Aa	63±7Aa
Hexanal	525±6Ad	547±18Cb	545±38Cb	574±18Cab	607±6Ca
(E)-2-Hexenal	24697±73Aa	24329±507A	24172±290A	24048±148Aa	24030±155Aa
(E,E)-2,4-Hexadienal	96±0Aa	93±3Aa	93±1Aa	91±4ABa	88±5Aa
Sum of C_5-C_6 aldehydes	24860±73Aa	24485±507A	24331±290A	24205±148Aa	24180±155Aa
Heptanal	56±2Ac	53±5Bc	88±0Ab	72±2Aa	74±2Ba
(E)-2-Heptenal	91±3Ac	94±2Bc	93±1Cc	106±0.1Bb	117±3Ca
(E,E)-2,4-Heptadienal	55±1Ac	54±1ABb	60±3ABa	57±0.1Cb	66±6Ca
Nonanal	1636±5Ab	1638±19Aa	1639±34Aa	1646±1Ba	1654±17Aa
Sum of C_7 - C_9 aldehydes	1839±6Ab	1839±19Abb	1881±34Aa	1882±2Ca	1911±18Ca
Alcohols					
1-Pentanol	41±1Aa	44±5Aa	42±2Aa	43±4Aa	41±2Aa
1-Penten-3-ol	268±0Aa	258±1Abc	261±2Ab	258±1Abc	255±2Ac
(E)-2-Penten-1-ol	43±1Aa	44±4Aa	38±3Aa	39±2Aa	40±0Aa
(Z)-2-Penten-1-ol	360±2Aa	354±2Aa	351±11Aa	330±13ABb	292±4Bc
1-Hexanol	606±8Aa	592±12Aa	561±8Bb	564±9Ab	537±3Bc
(E)-2-Hexen-1-ol	1174±4Aa	1173±28Aa	1163±2Aa	1158±18Aa	1142±5Aa
(Z)-3-Hexen-1-ol	1625±3Aa	1617±7Aa	1618±25Aa	1611±67Aa	1602±39Aa
Sum of C_5-C_6 alcohols	4116±9Aa	4081±32Aa	4034±29Aab	4000±7171Abc	3909±39Ac
Esters					
Hexyl acetate	21±1Aa	21±3Aab	18±1Abc	16±1Ac	16±1Ac

(Z)-3-Hexenyl Acetate	189±0Aa	168±8Ab	160±1Abc	159±1Abc	151±9Ac
Sum of C_6 esters	210±1Aa	189±8Ab	178±11Abc	175±2Abc	167±9Ac
Ketones					
3-Pentanone	237±7Aa	236±0Ac	278±0Aa	263±2Ab	264±3Ab
1-Penten-3-one	892±14Aa	811±1Ba	810±9Ba	811±5Ba	807±6Bb
6-Methyl-5-hepten-2-one	23±0Aa	20±0Bb	19±1Bb	19±1Bb	18±1Bb
Sum of C_5 - C_8 ketones	1153±16Aa	1066±11Bc	1107±9Ab	1092±5Ab	1089±6Bbc
			CPT		
Aldehydes					
Propanal*	59±1Ac	62±3Ab	64±4Ab	65.3±4Ab	71±3Ba
Pentanal	14±1Ac	15±1Bb	16±1Bb	20.5±2Aa	22±1ABa
(E)-2-Pentenal	67±0Aa	68±1Aa	68±3Aa	63±4Aab	60±2Ab
Hexanal	525±6Ad	584±6Bc	610±8Bbc	625±19Bab	638±9Ba
(E)-2-Hexenal	24697±73Aa	24574±103A	24366±141A	24156±381Aab	23790±159Ab
(E,E)-2,4-Hexadienal	96±0Aa	94±3Aa	92±0Aa	91±3Aa	87±1Ab
Sum of C_5-C_6 aldehydes	24860±73Aa	24735±103A	24525±141A	24313±381Abc	23937±159Ac
Heptanal	56±2Ac	55±1Bc	59±2Cc	63±1Bb	73±4Ba
(E)-2-Heptenal	91±3Ac	101±1Ab	100±1Bb	102±2Cb	125±0Ba
(E,E)-2,4-Heptadienal	55±1Ac	62±6Abc	63±4Abc	68±1Aab	76±2Ba
Nonanal	1636±5Ab	1642±3Aa	1638±26Aa	1669±5Aa	1673±36Aa
Sum of C_7 - C_9 aldehydes	1839±6Ad	1859±7Abc	1860±27Abc	1903±5Bab	1946±36Ba
Alcohols					
1-Pentanol	41±1Aa	42±3Aa	41±0Aa	39±1Aa	31±1Ab
1-Penten-3-ol	268±0Aa	266±15Aab	263±7Aab	251±8Aab	239±12Ab
(E)-2-Penten-1-ol	43±1Aa	42±1Aab	39±0Abc	37±1Ac	34±2Bc
(Z)-2-Penten-1-ol	360±2Aa	350±18Aa	337±10Aab	316±18Bbc	298±11Bc
1-Hexanol	606±8Aa	601±22Aa	592±5ABb	579±19Ab	552±3ABc
(E)-2-Hexen-1-ol	1174±4Aa	1176±2Aa	1170±3Aa	1169±29Aa	1141±5Aa
(Z)-3-Hexen-1-ol	1625±3Aa	1600±11Aab	1582±14Aab	1575±21Abc	1537±28Ac
Sum of C_5-C_6 alcohols	4116±9Aa	4076±34Aab	4023±19Abc	3964±45Ac	3833±3Ad
Esters					
Hexyl acetate	21±1Aa	19±2Aab	16±0Abc	14±1Ac	12±1Bc
(Z)-3-Hexenyl Acetate	189±0Aa	164±3Ab	152±3Bc	149±1Bc	145±3Ac
Sum of C_6 esters	210±1Aa	182±4Ab	168±3Bc	163±2Bcd	158±3Bd
Ketones					
3-Pentanone	237±7Aa	236±9Aab	229±8Bbc	210±13Bc	206±7Bc
1-Penten-3-one	892±14Aa	883±1Aa	878±5Aa	875±8Aa	873±11Aa
6-Methyl-5-hepten-2-one	23±0Aa	23±1Aa	21±0Abc	22±2Ab	19±1Bc
Sum of C_5 - C_8 ketones	1153±16Aa	1142±9Aa	1127±9Aab	1107±15Ab	1099±13Ab

Results are expressed as the mean ± standard deviation of two independent determinations (n = 2). Different uppercase letters (A-C) in the column indicate statistically significant ($p \leq 0.05$) differences among different EVOOs packaging at the same storage time. Different lowercase letters (a-d) in the row indicate statistically significant ($p \leq 0.05$) differences within the same EVOO packaging during storage. C_5-C_6 Aldehydes (as expressed as the sum of pentanal, (E)-2-pentenal, hexanal, (E)-2-hexenal, and (E,E)-2,4-hexadienal), C_7-C_9 Aldehydes (as expressed as the sum of heptanal, (E)-2-heptenal, (E,E)-2,4-heptadienal, and nonanal), C_5-C_6 Alcohols (as expressed as the sum of 1-pentanol, 1-penten-3-ol, (E)-2-penten-1-ol, (Z)-2-penten-1-ol, 1-hexanol, (E)-2-hexen-1-ol, and (Z)-3-hexen-1-ol) C_6 Esters (as expressed as the sum of hexyl acetate and (Z)-3-hexenyl acetate), C_5-C_8 Ketones (as expressed as the sum of 3-pentanone, 1-penten-3-one, and 6-methyl-5-hepten-2-one).

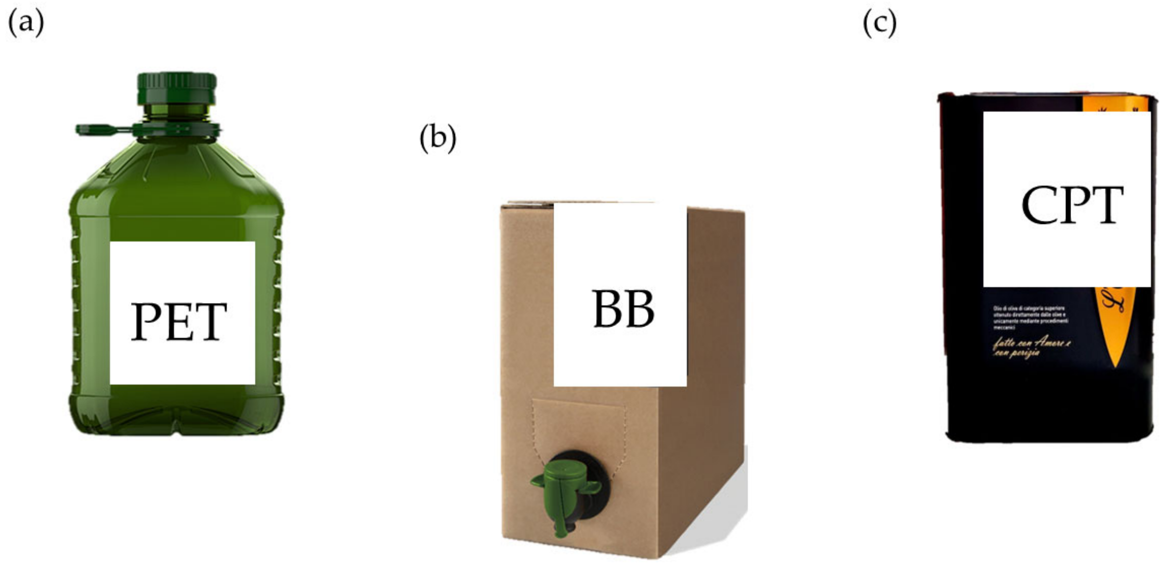

Figure S1. EVOO packaging materials: (a) green food-grade polyethylene terephthalate (PET) container (PET packaging); (b) bag-in-box (BB) system consisting of a multilayer pouch placed inside a parallelepiped-shaped corrugated paperboard box (BB packaging); and (c) tin-plated parallelepiped can with an internal varnish based on chrome-plated stripe coating (CPT packaging).
